# TFE3-Rearranged Renal Cell Carcinoma With Osseous Metaplasia: A Case Report of a Rare Entity With an Unusual Finding

**DOI:** 10.7759/cureus.73072

**Published:** 2024-11-05

**Authors:** Raghad A Bokhari, Ali S Al-Zughbi, Ziyad A Bokhari

**Affiliations:** 1 Pathology and Laboratory Medicine, King Fahad Medical City, Riyadh, SAU; 2 Pathology and Laboratory Medicine, King Saud Bin Abdulaziz University for Health Sciences College of Medicine, Riyadh, SAU

**Keywords:** mit family, osseous metaplasia, pediatric, renal cell carcinoma, tfe3

## Abstract

*TFE3*-rearranged renal cell carcinoma (RCC) is a rare subtype of renal tumor that typically affects the pediatric age group. Previous exposure to chemotherapy agents increases the possibility of having this subtype. Morphologically, it displays a broad spectrum of growth patterns, but it can be distinguished by the presence of melanin pigment or psammoma bodies. Osseous metaplasia is an uncommon morphological finding in this particular subtype, and it has been reported in two cases in the literature. We describe the third case of *TFE3*-rearranged renal cell carcinoma with osseous metaplasia in a healthy 13-year-old male, presenting with worsening abdominal pain and hematuria.

## Introduction

Renal cell carcinoma (RCC) is responsible for 2% of cancer cases worldwide and 2% of all cancer-related fatalities [1]. Microphtalmia-associated transcriptional factor (MiT) family translocation RCC involves *TFE3* or *TFEB* gene fusion to various genes at several different chromosomal locations [2]. This subtype makes up about 40% of pediatric RCCs and 1.6%-4% of adult RCCs. Osseous metaplasia is an unusual finding in renal tumors, but it is documented in association with oncocytic renal tumors such as chromophobe renal cell carcinoma (CCRCC) and others like clear cell RCC (CCRCC) and papillary RCC (PRCC). The literature describes two cases of TFE3-rearranged RCC with osseous metaplasia [3,4].

Herein, we report the third case of *TFE3*-rearranged RCC with osseous metaplasia proved by immunohistochemistry (IHC), with a short interval between symptoms and definitive surgery.

## Case presentation

A 13-year-old male presented to the emergency department complaining of progressive abdominal pain at the right lower quadrant for the last couple of days, associated with low-grade fever, vomiting, diarrhea, and hematuria. The patient was medically and surgically free with no family history of cancer. Abdominal examination revealed abdominal tenderness at palpation in the right lower quadrant. Ultrasound imaging for both kidneys showed a well-demarcated right kidney lesion, measuring 11.4 x 11.3 x 9.5 and showing mixed echogenicity, hyperechoic wall, and central vascularity and calcification. The left kidney was unremarkable. A computed tomography (CT) scan showed a right kidney exophytic necrotic mass. There was no evidence of invasion into the adjacent structures or distant metastasis. Radiologically, the differential diagnosis was Wilm's tumor versus RCC.

**Figure 1:**
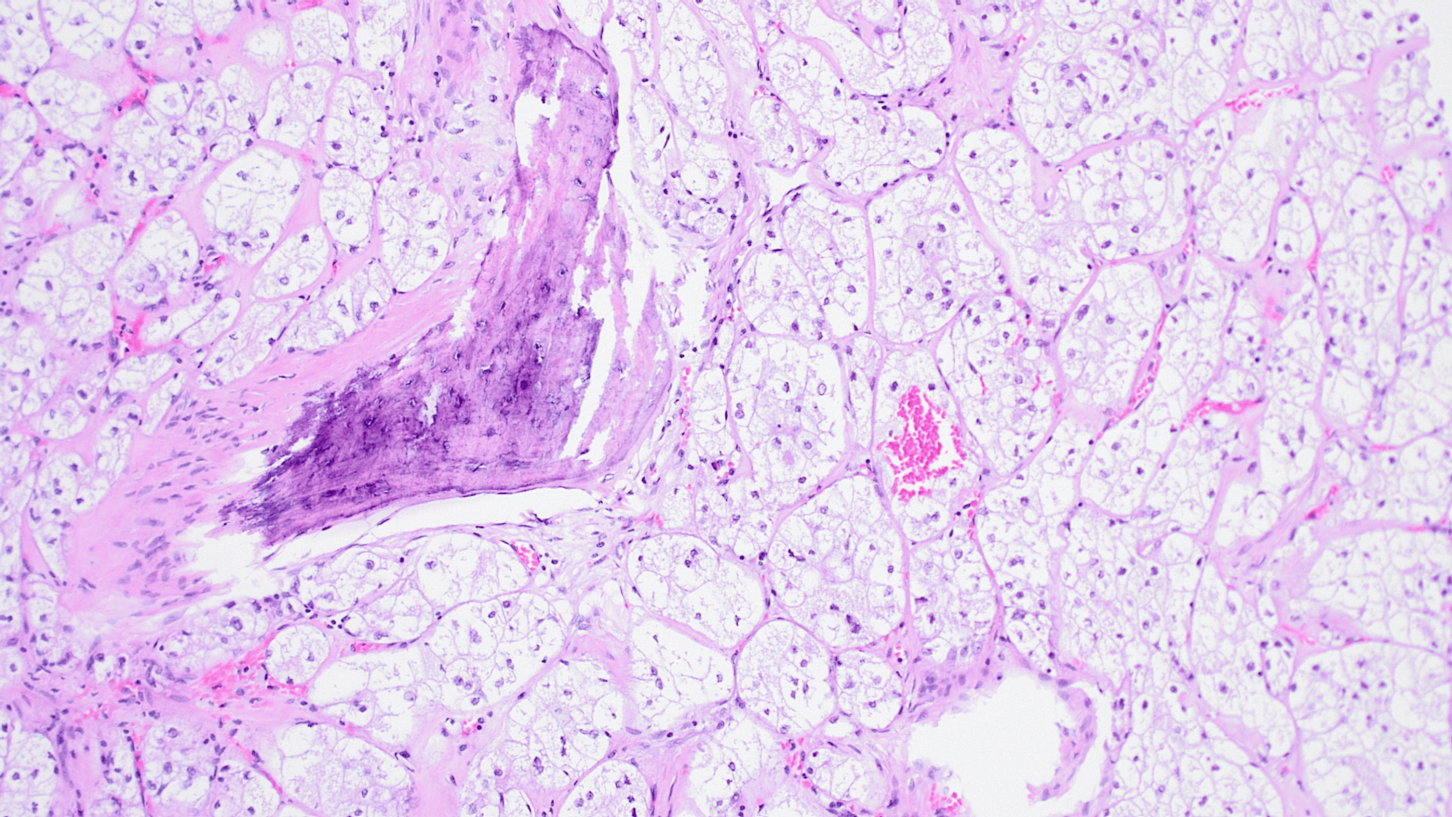
A photomicrograph showing nests of neoplastic cells with abundant clear cytoplasm and focal ossification Hematoxylin and Eosin stain x200 magnification

**Figure 2:**
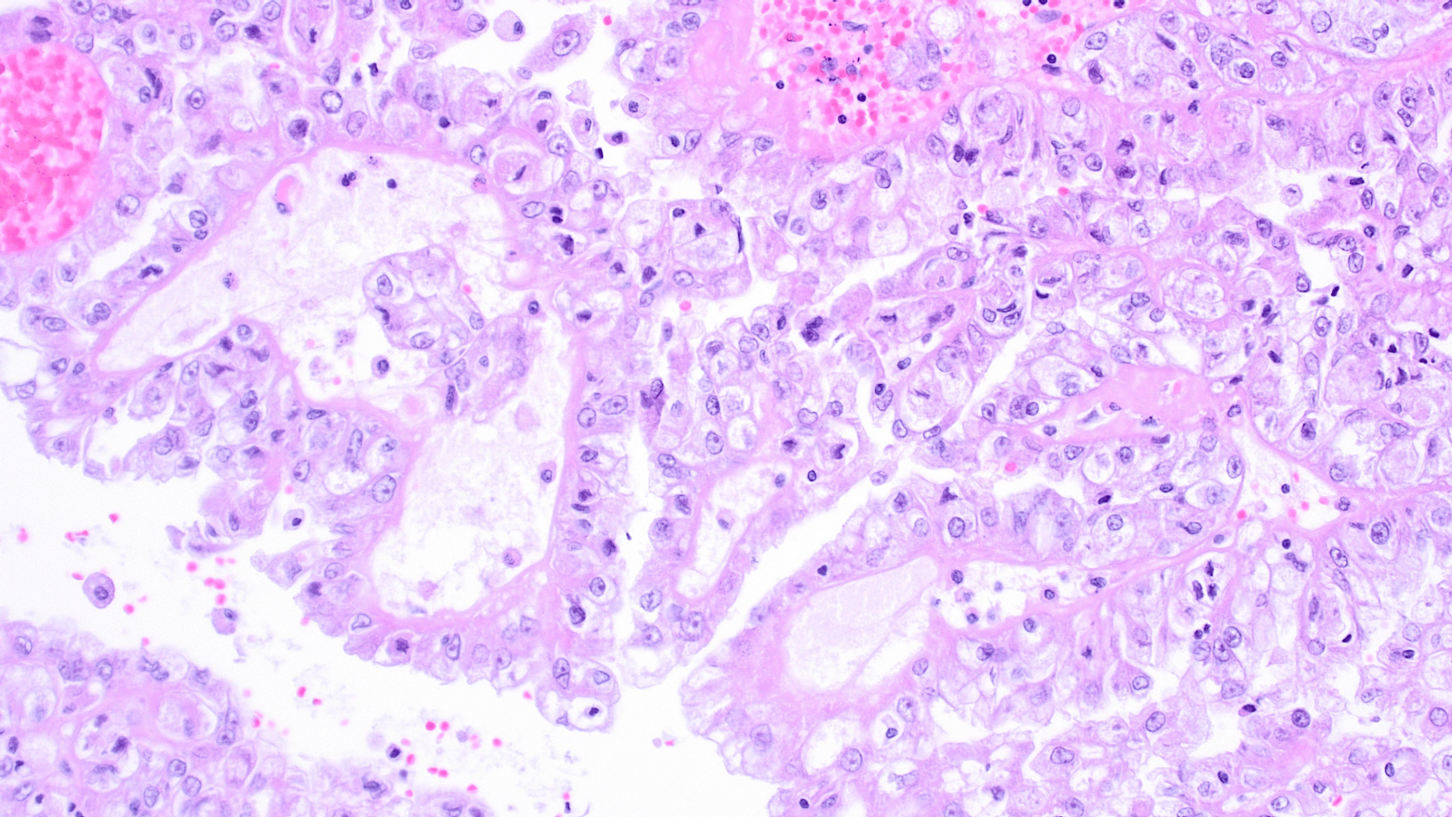
A photomicrograph showing the focal papillary area formation Hematoxylin and Eosin stain x200 magnification

**Figure 3:**
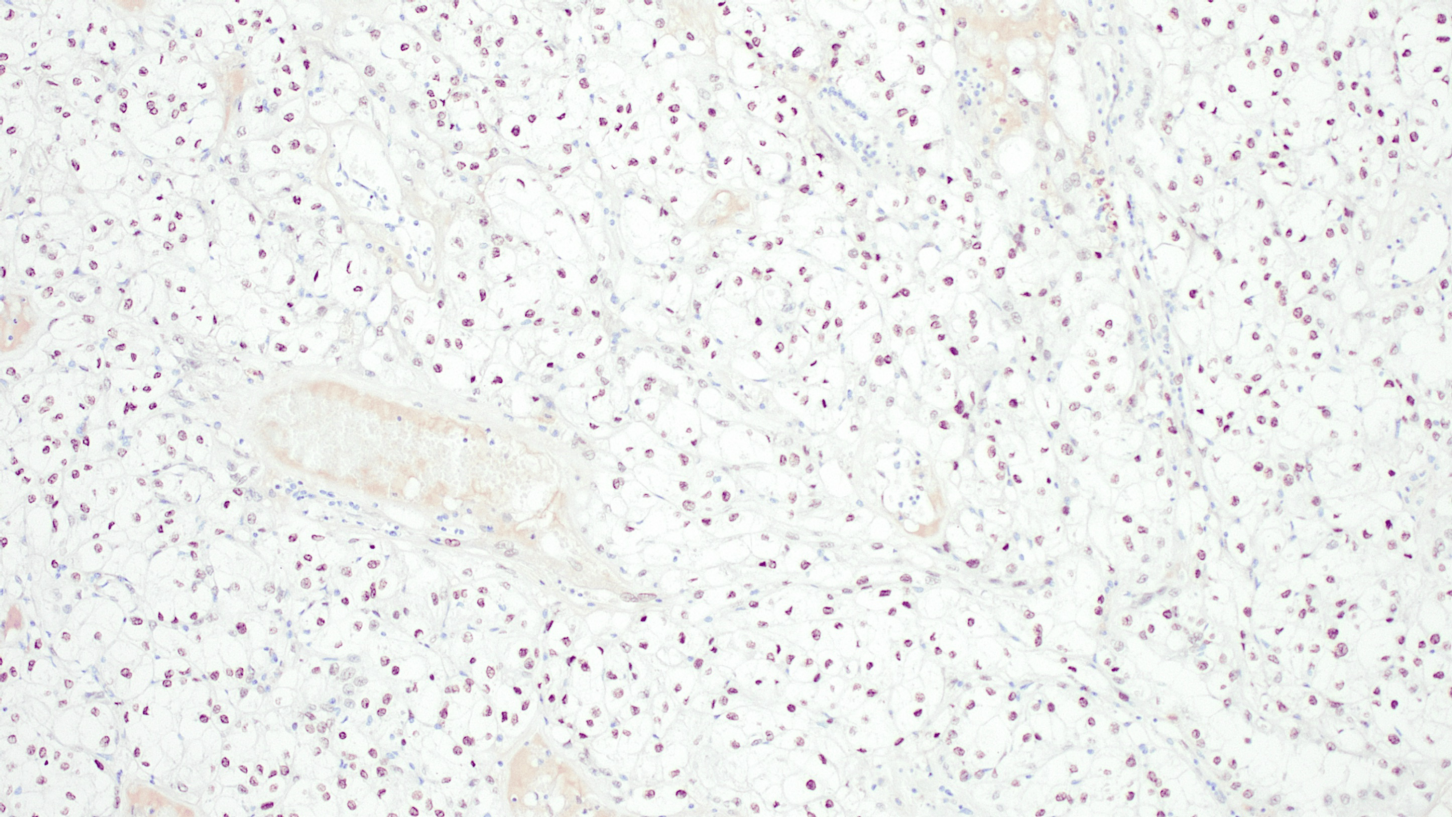
TFE3 immunohistochemical stain showing a nuclear stain of tumor cells *TFE3*, X100 magnification

The patient underwent radical nephrectomy. Macroscopically, there was a well-defined, friable yellow mass measuring 11.0 x 7.5 x 7.0 cm involving 75% kidney parenchyma. The tumor invaded the renal sinus and did not invade the perirenal fat (pT3a). Microscopically, the tumor was composed of neoplastic cells arranged in a predominantly solid growth pattern, with a focal area of pseudopapillary architecture. The cells contain voluminous clear cytoplasm and the nucleoli were visible at x100 (World Health Organization/International Society of Urological Pathology (WHO/ISUP) grade 3). In addition to focal areas of metaplastic bone formation, psammoma calcification, and melanin pigment (Figures 1, 2). A panel of immunohistochemical stains was done and tumor cells showed nuclear staining for *TFE3*, granular cytoplasmic staining for AMCAR, as well as focally positive for HMB45, and negative staining for CK7 and EMA (Figure 3). The diagnosis rendered was *TFE3*-rearranged renal cell carcinoma with osseous metaplasia. After an unremarkable postoperative course, our patient was discharged on day six and scheduled for a follow-up appointment at the outpatient department.

## Discussion

RCC accounts for 2% to 6% of all pediatric renal tumors, and the most common subtype in this age group is MiT-RCC [1]. In 2016, the World Health Organization defined MiT-RCC as a distinct entity, and it includes Xp11 translocation RCC with *TFE3* gene fusions and t(6;11) RCC with the *TFEB *gene [5]. The most recent edition of the WHO classification divided RCCs into those that were determined by morphology and molecular characteristics. ELOC (formerly TCEB1)-mutated RCC, SMARCB1 (INI1)-deficient RCC, *TFE3*-rearranged RCCs, *ALK*-rearranged RCCs, and *TFEB*-rearranged and *TFEB*-amplified RCCs, are examples of molecularly characterized RCCs [1]. Previous exposure to cytotoxic chemotherapy is a risk factor for this tumor subtype. Patients with Xp11 translocation RCCs have a survival rate lower than that of patients with papillary RCCs and comparable to that of patients with clear cell RCCs [1].

Histologically, *TFE3*-rearranged RCC can mimic other renal cell neoplasms including clear cell renal cell carcinoma, papillary renal cell carcinoma, and clear cell papillary renal cell tumor. This diagnosis is suspected when the tumor comprises various architectural patterns, including solid, papillary, pseudopapillary, and cystic, and when the neoplastic cells contain abundant clear cytoplasm. Other distinctive features include psammoma body calcification and melanin pigment. This tumor is usually positive for *Pax8*, melanocytic markers (melan-A and HBM-45), and cathepsin-K. The essential feature to diagnose this entity is robust nuclear labeling for *TFE3* in a clean background using immunohistochemistry (IHC), *TFE3* rearrangement detected by using break-apart fluorescence in microscopy (FISH), or *TFE3* gene fusion detected by RNA sequencing [1].

Osseous metaplasia is defined as the presence of either mature or immature bone in tissue that isn’t normally composed of bone, despite the lack of a well-defined ossification process. It was proposed that osseous metaplasia within a tumor might be secondary to hyalinization, ischemia, hemorrhage, necrosis, or fibrosis [6]. In renal tumors, this phenomenon has been well-documented, with multiple reports of occurrences in CCRCC, PRCC, and chromophobe RCC [3]. There is still a lack of knowledge regarding the prognostic implications of osseous metaplasia. It is crucial to differentiate osseous metaplasia from sarcomatoid carcinoma with bone formation (osteosarcomatous differentiation), as the latter typically has a poor prognosis [6]. In the literature, there have been two reports of *TFE3*-rearranged RCC with osseous metaplasia, one in an adolescent and the other in an elderly woman [3,4].

## Conclusions

In conclusion, *TFE3*-rearranged renal cell carcinoma is the most common renal cell carcinoma in children. To diagnose this specific subtype, additional studies, such as IHC stain or FISH, are required for confirmation. This case study provides more information on a unique pathological feature of *TFE3*-rearranged RCC and highlights the need for more research to fully understand the prognosis of these tumors in association with osseous metaplasia. In addition, further research into the underlying processes of osseous metaplasia within RCC and its prognostic relevance will be worthwhile.
